# Anti-Obesity Medications Versus Metabolic Bariatric Surgery as Pathways to Heart Transplant Eligibility in LVAD-Supported Patients: A Narrative Review

**DOI:** 10.3390/jcm15197489

**Published:** 2026-09-26

**Authors:** Noah Sicouri, Vanesa Brecher, Vasiliki Androutsopoulou, Maximiliano Brizzio, Prokopis-Andreas Zotos, Massimo Baudo, Serge Sicouri

**Affiliations:** 1 Department of Neuroscience, University of Pittsburgh, Pittsburgh, PA 15260, USA; nps67@pitt.edu; 2 Department of Cardiac Surgery Research, Lankenau Institute for Medical Research, Wynnewood, PA 19096, USA; vb4075@pcom.edu (V.B.); brizzioma@mlhs.org (M.B.); massimo.baudo@icloud.com (M.B.); 3 Department of Cardiothoracic Surgery, University of Thessaly, Biopolis, 41110 Larissa, Greece; androutsopoulouvasiliki@uth.gr (V.A.); zotospro@hotmail.com (P.-A.Z.)

**Keywords:** left ventricular assist device, heart transplantation, obesity, bariatric surgery, sleeve gastrectomy, GLP-1 receptor agonist, semaglutide, bridge to candidacy, transplant listing, body mass index

## Abstract

Obesity is a common and potentially modifiable barrier to heart transplant candidacy in patients supported by a durable left ventricular assist device (LVAD). Approximately 15% of LVAD recipients carry a body mass index (BMI) of 35 kg/m^2^ or greater; the 2024 International Society for Heart and Lung Transplantation guideline considers weight loss to a BMI below 35 kg/m^2^ reasonable before listing, within an individualized assessment, and only 18.6% of these patients achieve a 10% weight reduction through supportive care alone. Two interventions can close this gap. Anti-obesity medications, principally glucagon-like peptide-1 receptor agonists (GLP-1 RAs), reduce BMI gradually without a procedural intervention; however, the largest LVAD series reports a BMI reduction from 38.3 to 36.2 kg/m^2^ at one year with 26% of patients who began above 35 kg/m^2^ falling below it, and efficacy depends on continued adherence and insurance coverage. In the FIGHT trial, liraglutide showed a non-significant, numerically unfavorable signal in recently hospitalized patients with advanced heart failure with reduced ejection fraction who were not on mechanical support. Metabolic bariatric surgery, most often laparoscopic sleeve gastrectomy, delivers a BMI reduction of 6 to 10 kg/m^2^ within 6 to 12 months and was associated with a threefold higher probability of subsequent transplantation in an observational Medicare cohort, at the cost of serious 30-day complications in 11.3% of LVAD recipients compared with 2.5% of non-LVAD bariatric patients. This narrative review compares mechanisms, LVAD-specific liabilities, and published outcomes of both strategies, including cardiovascular and arrhythmic outcomes, and reframes the choice as a trade-off between the speed of BMI reduction and the distribution of risk over time. We propose a hypothesis-generating selection framework, based on expert opinion, that considers excess BMI above threshold, expected time on device, bleeding profile, and gastrointestinal tolerance, and we define the evidence gaps that a prospective study should address.

## 1. Introduction

Durable left ventricular assist devices (LVADs) are implantable mechanical pumps that unload the left ventricle and restore systemic perfusion in patients with advanced heart failure who no longer respond to medical therapy. They extend survival and improve quality of life and, for a substantial proportion of recipients, function as a bridge to heart transplantation (HT) [1,2,3]. Ironically, the success of mechanical circulatory support has created a new clinical dilemma: patients now survive long enough for non-cardiac comorbidities to become the principal determinants of whether they ever undergo transplantation.

Obesity is a prominent example. The 2024 International Society for Heart and Lung Transplantation (ISHLT) guideline for the evaluation of transplant candidates states that, in candidates with a body mass index (BMI) of 35 kg/m^2^ or greater, weight loss to a BMI below 35 kg/m^2^ is reasonable before listing (Class IIa, Level of Evidence B-NR), and that bariatric surgery may be considered in selected patients depending on center expertise [4]. The threshold is therefore an anthropometric criterion applied within an individualized assessment of candidacy rather than an absolute contraindication. In the Interagency Registry for Mechanically Assisted Circulatory Support, 2620 of 17,095 recipients (15%) had a BMI of at least 35 kg/m^2^ at implantation [5]. Obesity in this setting is no longer a mortality signal so much as a morbidity amplifier as well as an access barrier: it raises the hazard of infection, device malfunction or thrombosis, arrhythmia, and readmission, and it can delay or preclude access to transplantation [5,6]. The window for correcting that barrier is finite. Bleeding, driveline infection, pump infection, stroke, and pump thrombosis accumulate with time on LVAD support; therefore, time spent pursuing weight loss carries its own clinical risk [6,7,8].

Two interventions can shorten that interval. Anti-obesity medications (AOMs), principally glucagon-like peptide-1 receptor agonists (GLP-1 RAs), produce clinically meaningful weight loss over months without any procedural insult [9,10]. Metabolic bariatric surgery (MBS) produces larger and more durable weight loss faster, at the cost of a major abdominal operation in an anticoagulated, preload-dependent patient [11,12]. The two options are rarely compared directly; to our knowledge, only one published cohort has evaluated both within the same LVAD population [13]. This narrative review compares AOMs and MBS as routes to transplant candidacy in LVAD-supported patients. We describe the mechanisms by which each strategy lowers BMI, their LVAD-specific liabilities, and the weight, transplant, and cardiovascular outcomes reported to date, and we reframe the decision as a trade-off between the speed of BMI reduction and the temporal distribution of risk. We conclude with a hypothesis-generating framework for patient selection and a research agenda to address the remaining evidence gaps.

## 2. Materials and Methods

This is a narrative review. No protocol was registered, and no formal risk-of-bias appraisal or quantitative pooling was performed. PubMed and Google Scholar were searched between June and August 2026 without a publication-year restriction. The core PubMed search string was (“left ventricular assist device” OR LVAD OR “mechanical circulatory support” OR “advanced heart failure”) AND (obesity OR “body mass index” OR “weight loss”) AND (“glucagon-like peptide-1 receptor agonist” OR semaglutide OR tirzepatide OR liraglutide OR “anti-obesity medication” OR “bariatric surgery” OR “sleeve gastrectomy” OR “gastric bypass” OR “heart transplantation” OR “bridge to candidacy”), adapted for Google Scholar. During revision, in September 2026, the search was updated and extended with the terms ventricular arrhythmia, atrial fibrillation, right ventricular failure, and cardiovascular outcomes. Reference lists of retrieved articles were screened for additional sources.

Records were screened by title and abstract, and potentially relevant articles were assessed in full text against predefined criteria. Studies were included if they were original reports (randomized trials, cohort studies, case series, or research letters with original data) or meta-analyses, enrolled adults supported by a durable LVAD, and reported weight or BMI, transplant listing or transplantation, cardiovascular, or safety outcomes after pharmacologic or surgical weight reduction. Because the LVAD-specific literature is limited, studies in broader advanced heart failure and general bariatric or obesity populations were retained when the underlying question did not depend on device physiology, and such evidence is identified as an extrapolation throughout. Conference abstracts without a full-text publication, pediatric studies, non-English publications, and single-patient case reports were excluded as outcome sources. Numerical values were extracted from the full texts and cross-checked against the original tables.

General bariatric and obesity cohorts were used specifically to characterize procedure-specific hazards such as bleeding under anticoagulation, marginal ulceration, and altered oral drug absorption, which have not been quantified in LVAD-supported groups. Mechanistic and review sources were selected for topical relevance. Weight change is reported as presented in the original studies, since investigators used different metrics, including absolute BMI change, absolute weight change, and percentage excess weight loss at differing follow-up intervals, which are not directly comparable. Cardiovascular outcomes, including arrhythmic events, heart failure events, invasive hemodynamics, and right ventricular function, were extracted when reported, and their LVAD-specific or extrapolated source is stated.

Generative artificial intelligence was not used to generate data, results, or interpretations. Any use was limited to language editing, and all authors reviewed and take responsibility for the content.

## 3. Obesity as a Barrier to Candidacy During LVAD Support

### 3.1. Why Weight Loss on Device Rarely Happens Spontaneously

Supportive care infrequently achieves clinically meaningful weight loss in this population. Among LVAD recipients with a BMI of at least 35 kg/m^2^, only 18.6% achieved a clinically significant weight loss of more than 10% during support, and the rate is no different between destination therapy and bridge-to-transplant strategies [5]. A contemporary synthesis reached the same conclusion, noting that fewer than 20% of supported patients reach a 10% reduction, and that this magnitude is typically insufficient to restore eligibility [6]. Device physiology may explain part of that failure. Continuous-flow pumps run at a fixed speed, so cardiac output rises only marginally during exertion, and peak oxygen uptake remains depressed after implantation. Maximal exercise capacity improves little, if at all, after LVAD placement, and pump speed adjustment has not reliably normalized exercise performance [14,15]. Patients therefore may be unable to achieve the level of energy expenditure on which most lifestyle-based weight loss programs depend.

### 3.2. The Cost of Not Crossing the Threshold

The consequence of remaining above a BMI of 35 kg/m^2^ is measurable. Among LVAD recipients with a BMI of 35 kg/m^2^ or greater at implantation, those who lost at least 10% of body weight were significantly more likely to undergo transplantation [5,12]. Conversely, patients who remain above the threshold continue to accrue device-related hazard without a clearly defined route to listing. This asymmetry, an accumulating risk on one side and an anthropometric threshold on the other, supports considering active intervention early, although the 2024 ISHLT guideline frames that threshold within an individualized assessment of candidacy [4]. Figure 1 illustrates the clinical problem and the two available pathways to transplant candidacy.

## 4. Anti-Obesity Medications

### 4.1. Mechanisms

Glucagon-like peptide-1 (GLP-1) is an incretin hormone secreted by L cells of the ileum and colon. It binds the GLP-1 receptor, activates G proteins and adenylyl cyclase, raises intracellular cyclic adenosine monophosphate, and enhances glucose-dependent insulin secretion from pancreatic beta cells [16]. Endogenous GLP-1 is short-lived and rapidly degraded by dipeptidyl peptidase-4, whereas therapeutic GLP-1 RAs are resistant to that enzyme and are long-acting [16,17].

Weight loss with GLP-1 RAs is driven primarily by reduced caloric intake, supported by delayed gastric emptying and increased satiety [16]. Receptors are expressed at two levels of the circuitry that governs feeding. In the hypothalamic arcuate nucleus, receptor activation increases anorexigenic pro-opiomelanocortin and cocaine- and amphetamine-regulated transcript signaling and suppresses orexigenic neuropeptide Y and agouti-related peptide signaling [16]. In the hindbrain, the area postrema lies outside the blood– brain barrier and allows circulating peptides to act directly on central satiety pathways; it relays to the adjacent nucleus tractus solitarius, which integrates that humoral input with vagal afferent signals from the gut to form the dorsal vagal complex [18]. Figure 2a summarizes this circuitry.

These effects are large enough to have supported regulatory approval for long-term weight management. Once-weekly semaglutide 2.4 mg produced a mean body weight change of −14.9% versus −2.4% with placebo at 68 weeks in adults with overweight or obesity [10], and model-based synthesis across placebo-controlled trials confirms a class effect with dose-dependent efficacy [9]. Gastrointestinal adverse effects, mainly nausea, vomiting, diarrhea, and constipation, are the dominant tolerability limitation, while serious adverse events remain uncommon [9,17].

### 4.2. LVAD-Specific Considerations

The efficacy data above come from ambulatory populations without mechanical support, and three device-specific issues qualify their transfer.

First, a numerical safety signal was observed with liraglutide in advanced heart failure. In the FIGHT trial, 300 patients recently hospitalized with heart failure and reduced ejection fraction were randomized to liraglutide or placebo. Liraglutide did not improve post-hospitalization clinical stability (mean rank 146 versus 156, *p* = 0.31). Deaths occurred in 19 patients (12%) versus 16 (11%), hazard ratio 1.10 (95% CI, 0.57–2.14), and heart failure rehospitalization in 63 (41%) versus 50 (34%), hazard ratio 1.30 (95% CI, 0.89–1.88) [19]. Neither difference reached significance, so the trial does not establish harm, but neither does it provide reassurance, and the numerical direction was unfavorable. The signal is specific to one molecule, liraglutide, in one population, recently hospitalized patients with advanced heart failure with reduced ejection fraction who were not on mechanical support. It should not be generalized to incretin therapy as a class, to semaglutide, or to dual GIP/GLP-1 receptor agonists, and no randomized trial of any of these agents has been conducted in LVAD recipients. The FIGHT trial excluded patients receiving or planned for LVAD therapy within 180 days and those on other mechanical support, so its applicability to LVAD recipients is only indirect. Continuous-flow physiology further limits extrapolation. In LVAD recipients, systemic output is determined largely by pump speed, preload, and right ventricular function rather than by native left ventricular contractility, so a drug effect on heart rate or left ventricular performance may not carry the same clinical consequence; conversely, the preload dependence of the pump creates a hazard, suction during volume depletion, that did not exist in the FIGHT population [20]. Patients on continuous-flow support are also substantially protected from the hemodynamic consequences of sudden cardiac death, which may attenuate the relevance of the signal, but caution remains warranted [12,19].

Second, gastrointestinal intolerance carries a device-specific consequence. Nausea, vomiting, reduced oral intake, and delayed gastric emptying may produce volume depletion. Continuous-flow LVADs are preload dependent, and hypovolemia predisposes to suction events in which the inflow cannula draws against the ventricular wall, an event associated with tachycardia and ventricular arrhythmia [20]. No published series has demonstrated that early gastrointestinal intolerance precipitates low-flow or suction events in LVAD recipients. The concern is mechanistic rather than empirical, and is concentrated in the dose-escalation phase, before meaningful weight loss has accrued, which is also the phase in which adherence is most fragile [21].

Third, delayed gastric emptying alters the rate and extent of absorption of co-administered oral drugs [21]. In a population that depends on tightly monitored vitamin K antagonist therapy, that pharmacokinetic effect is not trivial and argues for closer international normalized ratio surveillance during initiation and titration.

### 4.3. Published Outcomes in LVAD Recipients

In the largest single-center experience, 44 of 333 LVAD recipients (13.2%) started a GLP-1 RA between 2018 and 2023; 4 who had started therapy more than one year before implantation were excluded, leaving 40 (12.0% of the cohort) for analysis, including semaglutide in 90% of patients. At one year, weight fell from 116 kg (98–134) to 110 kg (91–129), *p* < 0.001, and BMI from 38.3 kg/m^2^ (33.3–45.4) to 36.2 kg/m^2^ (31.4–41.2), *p* < 0.001, a median reduction of 6 kg. Glycated hemoglobin fell from 6.4% to 5.7% (*p* = 0.003) and N-terminal pro-B-type natriuretic peptide from 810 to 732 pg/mL (*p* = 0.04), with non-significant trends toward higher mixed venous oxygen saturation (66% to 68%, *p* = 0.090) and lower pulmonary vascular resistance (1.91 to 1.42 Wood units, *p* = 0.067). For heart transplant candidacy, the relevant figure is narrower. Twenty-seven patients (67.5%) began above 35 kg/m^2^, and 26% of these patients reached a BMI below 35 kg/m^2^ at one year [22]. The numerator was not reported; 26% corresponds to approximately 7 of the 27 patients and cannot refer to the full cohort of 40, in which no whole number of patients yields 26%.

No new safety signal emerged within the limits of a 40-patient series. Among the 40 patients, heart failure hospitalization occurred in 6 (15.0%), driveline infection in 5 (12.5%), hemocompatibility-related adverse events in 3 (7.5%), gastrointestinal bleeding in 2 (5.0%), and ischemic stroke in 1 (2.5%). One pump thrombosis was attributed to electrostatic interference rather than to therapy, and the single death (1 of 40, 2.5%) followed HeartMate II malfunction. The specific concern that rapid loss of body mass would displace the inflow cannula was not borne out: the absolute cannula coronal angle changed by 7 degrees (3.8–11.7), 21% of patients changed by more than 10 degrees, and regression against weight change gave a slope of 0.051 degrees per kilogram (*p* = 0.54). No patient stopped therapy because of an adverse event. Three patients (7.5%) stopped because of cost and insurance non-coverage, the leading reason for discontinuation and a concrete argument for confirming coverage before committing to a pharmacologic pathway [22].

Three further cohorts extend these observations. A single-center series of 21 LVAD recipients with class I to III obesity reported a median weight change of −12.4 kg (−20.0 to −2.9), equivalent to −7.4% of body weight (−16.3 to −2.1), over a median of 12 months on therapy; 17 of 21 lost weight, 9 of 21 required a pump speed reduction, and no major adverse event was attributed to treatment [23]. In a propensity-matched analysis of 1036 LVAD recipients drawn from a federated network of 98 healthcare organizations, GLP-1 RA use was associated with more frequent transplantation than non-use among matched patients (18.9% versus 8.5%; adjusted odds ratio 2.51; 95% CI, 1.72–3.67) and with fewer acute heart failure events (55.6% versus 68.9%; adjusted odds ratio 0.57) and fewer all-cause hospitalizations (62.5% versus 75.3%; adjusted odds ratio 0.55), with no difference in mortality, stroke, or cardiac arrest [24]. A second propensity-matched analysis of the same network, restricted to 527 obese recipients with 141 matched pairs, found that exposure was associated with a lower hazard of driveline infection at one year and, at three years, with lower all-cause mortality and higher rates of listing and transplantation [25]. Because both analyses draw on the same federated data source, they are not independent confirmations of each other, and neither can exclude healthy-user bias. Administrative and federated electronic health record data typically lack BMI trajectories, device settings, and the clinical reasons for prescribing, and patients able to maintain chronic self-injected therapy on LVAD support may differ systematically from non-users. Healthy-user bias, confounding by indication, and residual confounding may therefore explain part or all of these associations; neither analysis establishes that GLP-1 RA therapy causes higher transplantation rates or lower mortality.

The magnitude of BMI reduction remains a principal limiting factor. A reduction of roughly 2 kg/m^2^ over one year may be sufficient for a patient at 36 or 37 kg/m^2^. It is unlikely to be sufficient for a patient at 45 kg/m^2^ within the interval of support that most patients can be expected to tolerate without a major device-related complication, and in the largest treated LVAD cohort, 26% of the patients who began above 35 kg/m^2^ were below it at one year [22]. Whether newer agents with greater efficacy, such as tirzepatide, change this ceiling in LVAD recipients has not been evaluated separately.

## 5. Metabolic Bariatric Surgery

### 5.1. Mechanisms and Procedure Selection

Metabolic bariatric surgery encompasses several operations, of which laparoscopic sleeve gastrectomy (LSG) and Roux-en-Y gastric bypass (RYGB) dominate contemporary practice. LSG reduces gastric volume by resecting the greater curvature whereas RYGB combines gastric restriction with intestinal bypass. Gastric balloons and adjustable gastric banding have been largely abandoned: bands fail and require revisional operations at high rates, and balloons are limited by intolerance and early removal [26,27,28].

Neither operation works by mechanical restriction alone. Reduced reservoir capacity limits meal size and brings forward distension-mediated afferent signaling, which lowers total caloric intake [29]. Gut vagal afferents transduce both mechanosensory and chemosensory signals, and postprandial gastric distension activates stretch receptors that project centrally [18]. In parallel, resection of the gastric fundus during LSG substantially lowers circulating ghrelin, the principal orexigenic hormone, in both rodents and humans, whereas ghrelin levels after RYGB remain variable and inconsistent [30,31]. Postprandial GLP-1 and peptide YY rise after both operations [31]. Dietary preference, satiety, and food reward are all altered [29]. The mechanistic convergence and divergence of metabolic bariatric surgery with pharmacologic therapy is shown in Figure 2.

### 5.2. LVAD-Specific Considerations

Anticoagulation dominates the surgical risk calculus. Continuous-flow support generally requires chronic anticoagulation to prevent pump thrombosis, and every staple line and anastomosis becomes a bleeding surface [32]. In the 2023 Metabolic and Bariatric Surgery Accreditation and Quality Improvement Program dataset, 133 of 180,544 patients undergoing primary LSG or RYGB had an LVAD. Therapeutic anticoagulation was present in 45.9% of LVAD patients versus 3.0% of the remainder. LVAD patients had longer operative times (105.3 versus 84.6 min, *p* < 0.001), more serious complications (11.3% versus 2.5%, *p* < 0.001), more bleeding (5.3% versus 0.8%, *p* < 0.001), more readmissions (13.5% versus 2.9%, *p* < 0.001), and longer hospital stays (4.9 versus 1.2 days, *p* < 0.001). After adjustment, both LVAD status (odds ratio 2.83; 95% CI, 1.62–4.97) and RYGB (odds ratio 1.71; 95% CI, 1.60–1.83) remained independently associated with serious complications [33]. Across general bariatric cohorts, anticoagulant therapy is strongly associated with postoperative bleeding [32,34].

Procedure choice follows from that finding. LSG accounted for 73.7% of LVAD cases in the national dataset and 93.9% in the Medicare cohort, and RYGB independently increases risk. The principal advantage of LSG is the absence of gastrojejunal and jejunojejunal anastomoses and therefore the absence of marginal ulceration at those sites [35]. Bleeding marginal ulcers are the most common cause of delayed hemorrhage after RYGB, with a reported prevalence between 0.6% and 16% [36,37]. Recognized risk factors include smoking, diabetes, corticosteroid use, non-steroidal anti-inflammatory drug exposure, immunosuppression, larger pouch size, circular stapled anastomoses, and non-absorbable sutures [35,38]. These figures are derived from bariatric cohorts without LVADs and are extrapolations; no study has reported the incidence of marginal ulceration in LVAD recipients. The closest available population is patients on chronic anticoagulation. Among 132 anticoagulated patients followed for a mean of 5.2 years, long-term bleeding occurred in 18.3% after RYGB and 4% after sleeve gastrectomy (*p* = 0.017), and a bleeding marginal ulcer, seen in 13.4% of RYGB patients, was the most common cause, and 84.2% of bleeding events occurred on warfarin [32]. Some of the recognized risk factors, such as corticosteroid exposure and immunosuppression, are relevant to patients approaching transplantation, which makes these estimates plausible for LVAD recipients but not directly transferable.

Altered drug absorption is the third consideration. Bariatric surgery changes gastric pH, resects absorptive segments, and alters gastric emptying and mucosal surface area [39]. The direction and magnitude of the effect are drug and procedure specific. Exposure to rivaroxaban and apixaban appears largely preserved, whereas warfarin dose requirements change after surgery [40]. The only LVAD-specific dataset addressing this question compared 48 LVAD recipients who underwent sleeve gastrectomy with 96 propensity-matched LVAD-only controls. Weekly warfarin requirements differed at 12 months (46.8 ± 18.6 versus 38.1 ± 21.4 mg, *p* = 0.05), warfarin dose per kilogram was lower in the surgical group until month 6, and time in therapeutic range was significantly lower at 8 weeks, 3 months, and 6 months, without any difference in bleeding or thromboembolic events [41]. Anticoagulation is therefore harder to control after surgery, and that instability should be planned for rather than discovered.

### 5.3. Published Outcomes in LVAD Recipients

The surgical literature is more extensive and consistently reported than the pharmacologic literature. In the Medicare cohort, 2798 of 14,417 LVAD recipients (19.4%) had at least class II obesity and 198 (7.1%) underwent bariatric surgery between 2012 and 2019, including sleeve gastrectomy in 93.9%. Follow-up BMI was available in 113 patients at a median of 275 days: median BMI fell from 42.5 to 34 kg/m^2^, a median loss of 9.5 kg/m^2^ (IQR, 5–14), with 69 of 113 patients reaching 35 kg/m^2^ or below and 35 reaching 30 kg/m^2^ or below. Follow-up BMI was therefore missing in 85 of 198 patients (43%); the 69 patients who reached the study’s threshold of 35 kg/m^2^ or below represent 61% of those with follow-up and at least 35% (69 of 198) of the full surgical cohort. Thirty-day major adverse cardiovascular events (MACE) occurred in 6.1% of patients and one-year mortality was 12.6%. Heart failure readmission burden fell from 3.01 to 0.62 admissions per 100 person-days (incidence rate ratio 0.20; 95% CI, 0.11–0.38). Thirty-seven patients (18.7%) underwent transplantation at a median of 371 days (IQR, 246–575) and 13 (6.6%) underwent explantation for recovery. In this observational analysis, bariatric surgery was associated with a threefold higher probability of transplantation (subdistribution hazard ratio 2.95; 95% CI, 2.09–4.17) but with no change in mortality (hazard ratio 0.88; 95% CI, 0.67–1.14; *p* = 0.2), and transplantation was inversely correlated with follow-up BMI (r = −0.31, *p* = 0.003) [42].

Timing relative to implantation was associated with outcome. In fact, 24 operations were performed during the LVAD admission and 174 at a median of 702 days afterwards (IQR, 383–1120). Median length of stay was 8 days (IQR, 6–13) when surgery followed a separate admission and 41 days (IQR, 28–55) when it was concurrent, and 30-day mortality was eight times higher in the concurrent group [42]. This aligns with the wider experience of non-cardiac surgery on device, in which elective procedures carried perioperative MACE in 7.1% of cases and an early mortality hazard of 2.65 (95% CI, 1.74–4.03) relative to LVAD recipients who did not undergo surgery [43]. Deferring bariatric surgery until the patient has recovered from implantation therefore appears preferable, with concurrent surgery reserved for exceptional circumstances, although the concurrent group was small and likely sicker, so confounding by illness severity cannot be excluded [42].

Single-center series are concordant. Six LVAD recipients undergoing LSG with heparin bridging had a mean pre-operative BMI of 41.4 kg/m^2^; four (67%) were listed and three (50%) were transplanted [44]. Twelve LVAD recipients undergoing sleeve gastrectomy at another center reduced mean BMI from 42.5 kg/m^2^ before surgery to 32.8 kg/m^2^ at transplantation, with 66.7% reaching a BMI below 35 kg/m^2^ and 33.3% transplanted at a mean of 17.9 months after surgery [45]. Among 15 patients with advanced heart failure and a BMI above 35 kg/m^2^, BMI fell from 39.8 kg/m^2^ (39.0–42.2) to 31.6 kg/m^2^ (27.7–35.3) at a median of 11 months with no surgery-related mortality, and post-transplant survival was comparable with 62 patients who did not undergo surgery [46]. In a matched cohort of patients with heart failure with reduced ejection fraction, sleeve gastrectomy produced a BMI change of −9.9 kg/m^2^ (95% CI, −12.2 to −7.6) versus −2.7 kg/m^2^ in controls, with improvement in ejection fraction and functional class and a survival hazard ratio of 0.20 (95% CI, 0.07–0.62) [47].

A meta-analysis pooling eight studies and 59 LVAD recipients, 22 (37%) with concurrent and 37 (63%) with staged sleeve gastrectomy, found mean BMI fell from 46.7 kg/m^2^ (95% CI, 42.9–50.6) to 33.4 kg/m^2^ (95% CI, 30.2–36.6) at a mean follow-up of 12.7 months (*p* < 0.01). Sixty-six percent of patients were listed and 33% were transplanted. Complication rates and survival did not differ significantly between the concurrent and staged approaches, but that comparison was underpowered, with the complication estimate for the concurrent group spanning 1% to 87%, and therefore should not be read as contradicting the timing signal in the Medicare data [48].

## 6. Direct Comparisons and Evidence Base

Only two studies have compared the two strategies within the same population. In a mixed retrospective and prospective series of 18 patients with heart failure and a BMI above 35 kg/m^2^, surgical patients lost 18.6 kg and 6.4 kg/m^2^ at six months compared with 1.9 kg and 0.7 kg/m^2^ in non-surgical patients. Ejection fraction rose 5.9% in the surgical group and fell 5.9% in the non-surgical group, without statistical significance [49]. In the only single-center comparison confined to LVAD recipients, 42 patients received obesity management medications and 7 underwent LSG. Baseline BMI was 42.7 versus 53.8 kg/m^2^. At three months, BMI fell by 0.9 versus 8.6 kg/m^2^, and total reduction reached 3.0 versus 15.9 kg/m^2^. Five of 42 medication-treated patients were listed and three transplanted, versus one of seven surgical patients listed and transplanted. Three deaths from heart failure progression occurred, and one postoperative death occurred within six months [13].

Two features of that comparison deserve emphasis. The surgical group started 11 kg/m^2^ heavier, which is the allocation pattern that clinical judgment would predict and which makes the two groups non-exchangeable. And the medication arm still reached listing more often in absolute terms, because it was already closer to the threshold. The comparison therefore describes how the strategies were allocated in practice rather than which is more effective. Table 1 assembles the published outcomes for both strategies, and Figure 3a displays the reported baseline and follow-up BMI of each cohort against the 35 kg/m^2^ threshold. Because populations, baseline BMI, follow-up intervals, and completeness of follow-up differ, these values are descriptive and are not comparative treatment-effect estimates.

## 7. Discussion

### 7.1. Speed of Weight Loss Versus Distribution of Risk

Both strategies achieve meaningful BMI reduction. What distinguishes them is not overall safety, but the timing of benefit and risk: one produces more rapid weight loss, whereas the associated hazards are concentrated at different points along the treatment course. These differences should therefore inform clinical decision-making. Metabolic bariatric surgery concentrates its risk. Serious complications occur in 11.3% of LVAD recipients within 30 days, four to five times the rate in non-LVAD bariatric patients, with bleeding emerging as the dominant complication [33]. After that window closes, risk reverts substantially to the background hazard of device support, and the patient carries a BMI reduction of 6 to 10 kg/m^2^ that is less dependent on continued adherence than pharmacologic weight loss, although weight regain can occur [42,46,47,50]. Anti-obesity medications invert this profile. They require no procedural intervention, although gastrointestinal intolerance and volume depletion are treatment-related risks, and they instead distribute a lower-grade exposure across a longer interval, during which the background hazard of bleeding, infection, stroke, and pump thrombosis continues to accumulate [7,8]. Figure 3b represents this trade-off schematically.

Neither profile is universally safer, and the two strategies are best viewed as distinct modalities suited to different baseline severities. The critical question is how much weight loss a patient requires and how long LVAD support can reasonably be expected to bridge the patient to that goal. Transplant associations reported for both strategies come from observational cohorts, in which healthier patients are more likely to be selected for either intervention, so they should not be read as treatment effects.

A patient at 36.5 kg/m^2^ needs approximately 1.5 kg/m^2^, a reduction within the range reported after one year of GLP-1 RA therapy in LVAD cohorts, in which about one quarter of patients who began above the threshold crossed it on that timescale [22]. A patient at 46 kg/m^2^ needs approximately 11 kg/m^2^, a reduction not reported in any published pharmacologic LVAD series but within the range reported after surgery [42,45]. These examples are illustrative; they rest on median or mean changes from small, non-comparable cohorts and do not predict the response of an individual patient.

### 7.2. Cardiovascular and Arrhythmic Outcomes

Weight and transplant access are not the only outcomes that matter in this population. Obesity in LVAD recipients is associated with a higher hazard of arrhythmia (hazard ratio 1.188), device malfunction or thrombosis (1.323), and infection (1.215) [5], and right heart failure affects up to 40% of recipients [7]. Ventricular arrhythmias and electrical storm are a recognized problem in advanced heart failure, and mechanical circulatory support is increasingly used to stabilize unstable patients, including during catheter ablation [51]; in LVAD recipients, volume-related suction events can themselves trigger ventricular arrhythmia [20]. A weight-reduction strategy could therefore help or harm through cardiovascular pathways that are independent of BMI change, and these outcomes should be weighed alongside weight loss when the two strategies are considered.

The LVAD-specific evidence on these outcomes is sparse and observational. After GLP-1 RA therapy, invasive hemodynamics were unchanged in the largest single-center series, with right atrial pressure 6 versus 5 mmHg (*p* = 0.962), mean pulmonary artery pressure 22 versus 19 mmHg (*p* = 0.922), pulmonary capillary wedge pressure 10 versus 9 mmHg (*p* = 0.868), and cardiac output 5.08 versus 5.31 L/min (*p* = 0.507) [22]. In the federated network analysis, GLP-1 RA use was associated with fewer acute heart failure events and with no difference in stroke or cardiac arrest, which occurred in 18 (3.5%) versus 17 (3.3%) matched patients (adjusted odds ratio 1.06; 95% CI, 0.54–2.08) [24]. In a separate series, 9 of 21 patients required a pump speed reduction during therapy, suggesting that loading conditions can change during weight loss [23]. After bariatric surgery, heart failure readmissions fell from 3.01 to 0.62 per 100 person-days and 13 of 198 patients (6.6%) underwent explantation for myocardial recovery, but the Medicare analysis contained no hemodynamic or echocardiographic data on right ventricular function [42]. No LVAD study of either strategy has reported ventricular arrhythmia, atrial fibrillation, or right ventricular failure as a prespecified outcome, and cardiac arrest in one database analysis is the only arrhythmia-related estimate identified [24].

Evidence from populations without mechanical support is larger but extrapolated. In the SELECT trial, which randomized 17,604 adults with established cardiovascular disease and overweight or obesity without diabetes, semaglutide reduced major adverse cardiovascular events (6.5% versus 8.0%; hazard ratio 0.80; 95% CI, 0.72–0.90), although discontinuation for adverse events was about twice as frequent (16.6% versus 8.2%) [52]. Meta-analyses of randomized trials have reported either no significant effect of GLP-1 RAs on atrial fibrillation in patients with type 2 diabetes (odds ratio 0.87; 95% CI, 0.71–1.05) [53] or a lower incidence with semaglutide (relative risk 0.58; 95% CI, 0.40–0.85) [54]. After bariatric surgery, predominantly observational data associate surgery with lower all-cause mortality (hazard ratio 0.55; 95% CI, 0.49–0.62), cardiovascular mortality (0.59; 0.47–0.73), and incident heart failure (0.50; 0.38–0.66), but not with a significant reduction in atrial fibrillation (0.82; 0.64–1.06) [55]. Less invasive endovascular approaches, such as bariatric embolization, have been proposed for patients at high surgical risk but have not been evaluated in LVAD recipients [56]. None of these data were generated in patients with continuous-flow support, 55% of whom already had atrial fibrillation at baseline in the largest GLP-1 RA cohort [22], and Table 2 therefore labels each cardiovascular domain by its evidence source.

### 7.3. A Structured Approach to Selection

The available evidence does not support a universal preference for either strategy, and it does not support treating them as mutually exclusive. Table 2 compares the two approaches across clinically relevant domains and labels the source of evidence for each, and Table 3 proposes a hypothesis-generating framework, based on expert opinion, for considering the initial strategy.

Three working principles, which should be regarded as hypothesis-generating rather than evidence-based rules, can be drawn from the available data. First, excess BMI above the threshold may be a useful anchor for the initial choice, because it influences whether a pharmacologic trajectory can plausibly reach the target within the interval of support a patient is expected to tolerate. Second, a pharmacologic trial may be considered for patients close to the threshold; the illustrative margin of 3 to 4 kg/m^2^ in Table 3 was derived by extrapolating the approximately 2 kg/m^2^ annual reduction observed in the largest LVAD series over 18 to 24 months [22], and a defined reassessment point, with surgical referral considered when the trajectory is inconsistent with the target, may prevent indefinite continuation. Third, early surgical evaluation may be considered for patients far above the threshold, because time spent on an unsuccessful pharmacologic trial is itself a hazard in a population whose device-related complications accrue with time on support [7,8]. These values have not been tested. The only within-population comparison included 49 patients with an 11 kg/m^2^ difference in baseline BMI between groups [13], and the two larger pharmacologic analyses draw on the same data source [24,25]. Any application should therefore remain within the individualized assessment of candidacy recommended by the 2024 ISHLT guideline [4].

Sequential and combined use deserves formal study. Pre-operative pharmacologic weight loss may reduce operative difficulty, and post-operative pharmacologic therapy may extend or defend surgical weight loss in patients who plateau above 35 kg/m^2^. Neither sequence has been evaluated in LVAD recipients, and each carries a plausible hazard, since delayed gastric emptying superimposed on a reduced gastric reservoir may amplify intolerance and volume depletion. Structured, stepwise treatment pathways have been called for in this population, and their absence is a major obstacle to consistent practice [6].

Values are drawn from non-comparable cohorts that differ in population, baseline BMI, follow-up interval, and completeness of follow-up, and they are not comparative treatment-effect estimates. Evidence base: LVAD-specific, data from LVAD recipients; advanced HF, data from patients with advanced heart failure without LVAD support; general obesity or bariatric, data from populations without heart failure or mechanical support; expert extrapolation, the authors’ inference from indirect evidence. aOR, adjusted odds ratio; BMI, body mass index; CI, confidence interval; HF, heart failure; HFrEF, heart failure with reduced ejection fraction; HR, hazard ratio; INR, international normalized ratio; LVAD, left ventricular assist device; MACE, major adverse cardiovascular events; RCT, randomized controlled trial; sHR, subdistribution hazard ratio.

The framework is hypothesis-generating and based on expert opinion informed by the evidence reviewed here; it has not been prospectively validated, and its numerical values are illustrative rather than decision thresholds. In every case the decision should be made by a combined heart failure, transplant, and bariatric team within an individualized assessment of candidacy [4]. BMI, body mass index; HF, heart failure; INR, international normalized ratio.

### 7.4. Evidence Gaps and a Research Agenda

Five gaps limit current practice. First, no prospective trial of any anti-obesity medication has been conducted in LVAD recipients. The pharmacologic evidence base consists of two small single-center series totaling 61 exposed patients and two propensity-matched analyses of the same federated network, which cannot be counted as independent [22,23,24,25]. Second, no study has quantified whether incretin-related gastrointestinal intolerance produces low-flow or suction events, despite a clear mechanistic pathway [20]. Third, the surgical literature reports transplant listing and transplantation but rarely reports the counterfactual, namely what happens to comparable patients managed without intervention, so selection bias remains unquantified. Fourth, no comparative effectiveness study has been powered to compare the two strategies at equivalent baseline BMI, which is the only comparison that would be clinically actionable. Fifth, cardiovascular outcomes beyond weight and transplantation, including ventricular arrhythmia, atrial fibrillation, right ventricular function, and heart failure hospitalization, have not been reported as prespecified outcomes for either strategy in LVAD recipients, so the cardiovascular benefits observed in populations without mechanical support cannot yet be assumed to apply.

A pragmatic multicenter registry capturing baseline BMI, strategy chosen, BMI trajectory, hemocompatibility-related adverse events, ventricular arrhythmia, atrial fibrillation, right ventricular function, heart failure hospitalization, listing, and transplantation could address much of this within a few years. A randomized trial restricted to patients within 4 kg/m^2^ of threshold, where equipoise is most plausible, appears feasible and could answer the narrower question that arises most often in practice.

### 7.5. Limitations

This is a narrative review and inherits the limitations of that design. The search used a defined search string and eligibility criteria (Section 2) but was not a systematic review; no PRISMA-based screening log or formal risk-of-bias appraisal was applied, and study selection ultimately reflects the authors’ judgment of relevance. The underlying literature is small, predominantly retrospective, and drawn from selected single-center cohorts. Follow-up intervals, baseline BMI, and outcome definitions differ between studies, so the values displayed in Figure 3a and Table 2 are descriptive, were not pooled, and are not comparative estimates. Several procedure-specific hazards, particularly marginal ulceration and altered drug absorption, are extrapolated from bariatric populations without mechanical support. The same applies to most cardiovascular and arrhythmic outcomes, which have been studied mainly in populations without LVAD support. Finally, the framework proposed in Table 3 is hypothesis-generating and based on expert opinion, and its numerical values are illustrative rather than validated thresholds.

## 8. Conclusions

LVAD support can sustain patients with advanced heart failure but it does not correct the obesity that often remains the principal obstacle to heart transplantation. Weight loss in this population is rarely achieved without intervention, and only 18.6% of patients with a BMI of at least 35 kg/m^2^ lose 10% of body weight on device [5]. The hazards of bleeding, infection, stroke, and pump thrombosis accumulate with time on support, so the interval available to correct BMI is finite [7,8].

Metabolic bariatric surgery reduces BMI by 6 to 10 kg/m^2^ within months rather than years and was associated with a threefold higher probability of transplantation in an observational cohort, at the cost of serious 30-day complications in 11.3% of LVAD recipients [33,42]. Anti-obesity medications require no procedural intervention, improve the cardiometabolic profile, and were associated with higher transplantation rates in matched observational cohorts, but deliver approximately 2 kg/m^2^ over a year, and 26% of patients who began above 35 kg/m^2^ in the largest LVAD series fell below it at one year [22,24].

The choice is therefore not between a safe option and a dangerous one, but between different patterns of risk. The key considerations are the amount of BMI reduction required, the timeframe in which it must be achieved, and whether risk is concentrated within a discrete procedural period or distributed across a longer duration of LVAD support. Excess BMI above the threshold, expected time on device, bleeding profile, arrhythmic and right ventricular status, and gastrointestinal tolerance may inform that decision, which belongs to a combined heart failure, transplant, and bariatric team working within the individualized assessment recommended by the 2024 ISHLT guideline [4]. Prospective LVAD-specific data on pharmacologic therapy, a comparative study at matched baseline BMI, and systematic reporting of cardiovascular and arrhythmic outcomes are the evidence the field most needs.

## Figures and Tables

**Figure 1:**
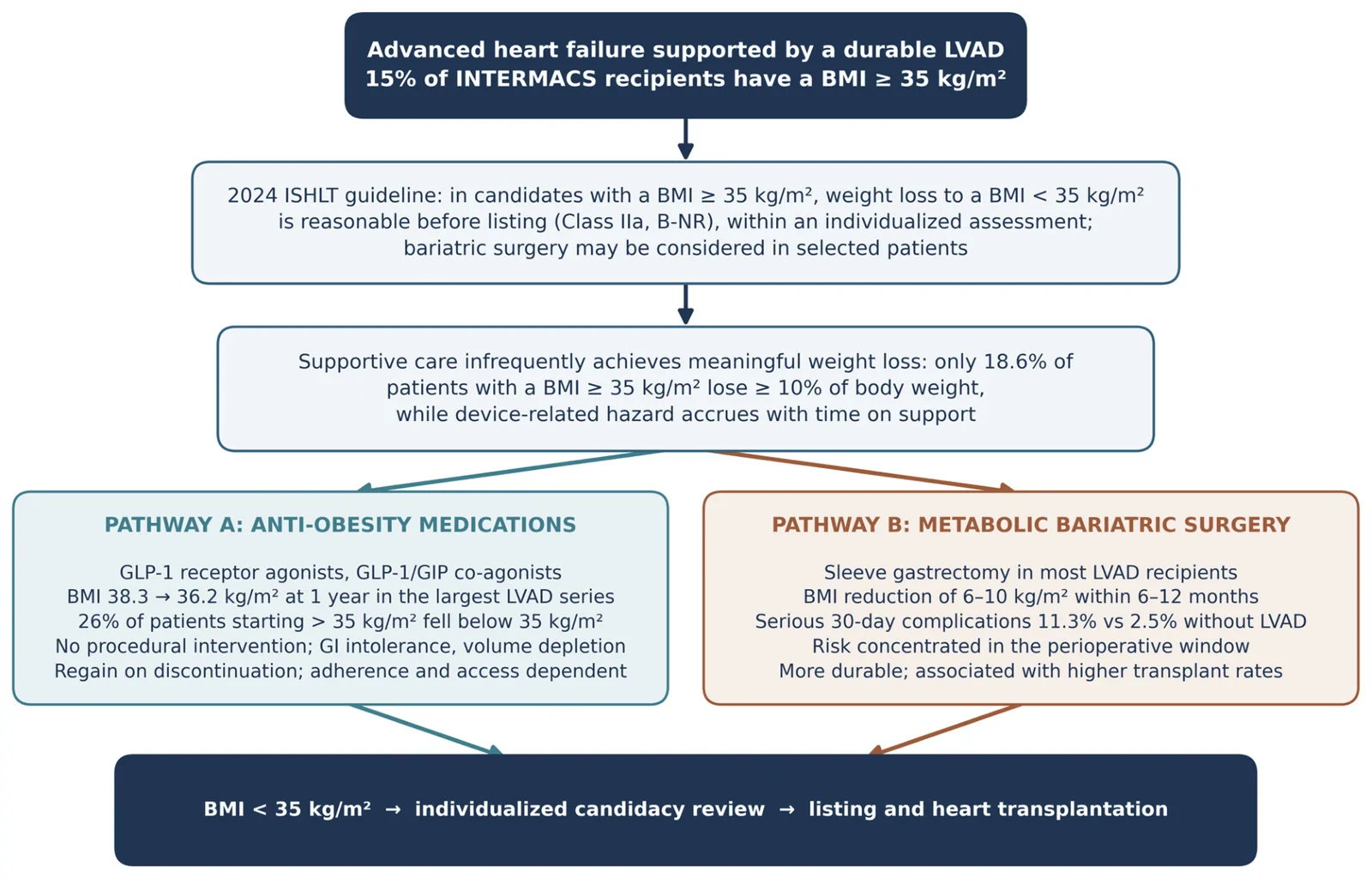
The clinical problem and the two available pathways to transplant candidacy in LVAD-supported patients with obesity. Supportive care infrequently achieves meaningful weight loss on device, so the decision often lies between a pharmacologic pathway that lowers BMI gradually without a procedural intervention and a surgical pathway that lowers BMI more quickly at the cost of concentrated perioperative risk. The 2024 ISHLT guideline considers weight loss to a BMI below 35 kg/m^2^ reasonable before listing, within an individualized assessment [4]. In the largest LVAD series, the 26% figure refers to patients whose baseline BMI exceeded 35 kg/m^2^. BMI, body mass index; GI, gastrointestinal; GIP, glucose-dependent insulinotropic polypeptide; GLP-1, glucagon-like peptide-1; INTERMACS, Interagency Registry for Mechanically Assisted Circulatory Support; ISHLT, International Society for Heart and Lung Transplantation; LVAD, left ventricular assist device. Created in BioRender. Sicouri, S. (2026) https://BioRender.com/om1nz18.

**Figure 2:**
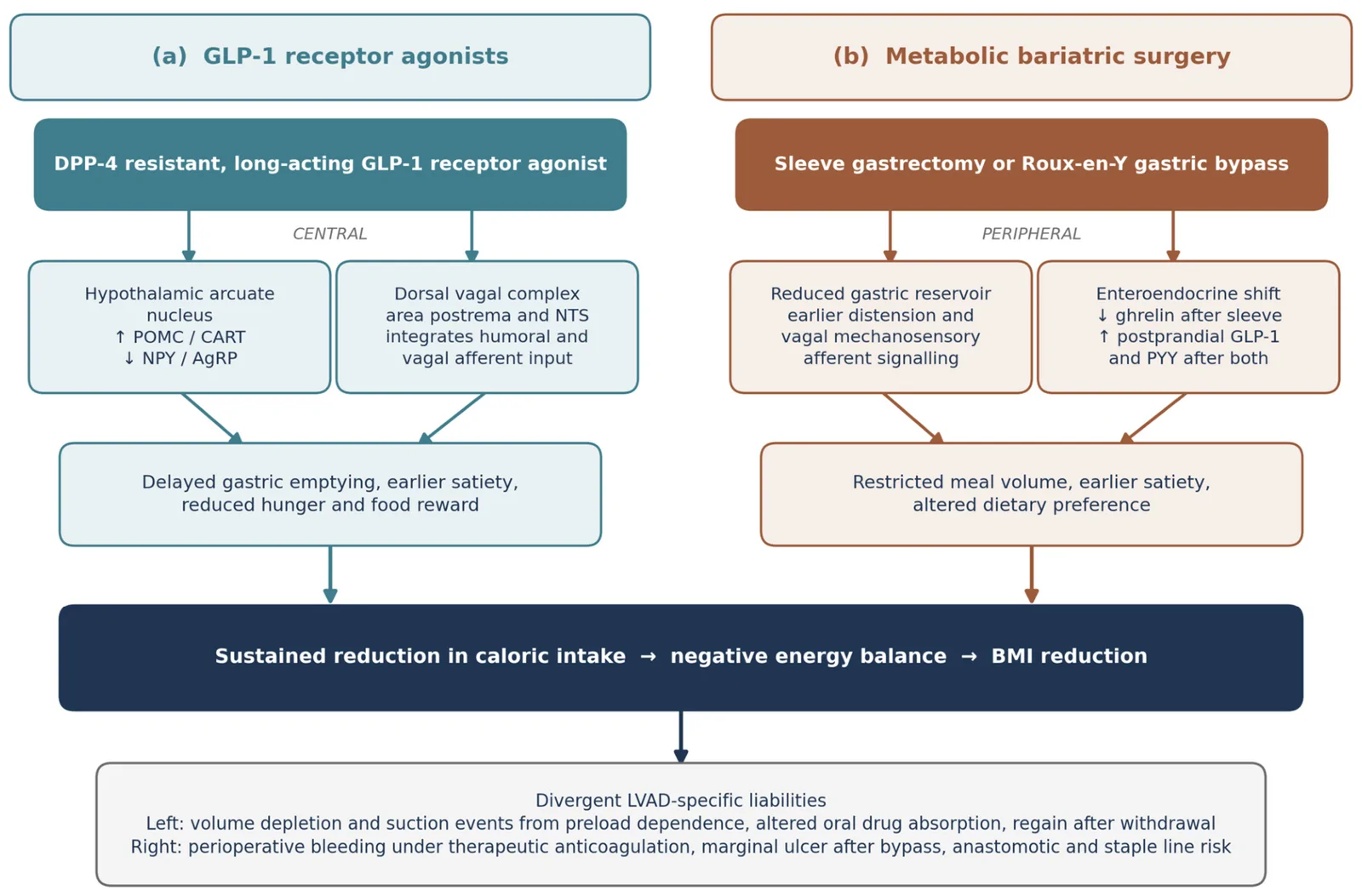
Convergent and divergent mechanisms of the two strategies. (**a**) GLP-1 receptor agonists act on the hypothalamic arcuate nucleus and the hindbrain dorsal vagal complex and delay gastric emptying. (**b**) Metabolic bariatric surgery reduces gastric reservoir capacity and shifts enteroendocrine signaling. Both converge on a sustained reduction in caloric intake, but their LVAD-specific liabilities differ. AgRP, agouti-related peptide; CART, cocaine- and amphetamine-regulated transcript; DPP-4, dipeptidyl peptidase-4; GLP-1, glucagon-like peptide-1; NPY, neuropeptide Y; NTS, nucleus tractus solitarius; POMC, pro-opiomelanocortin; PYY, peptide YY. Created in BioRender. Sicouri, S. (2026) https://BioRender.com/j3wscgs.

**Figure 3:**
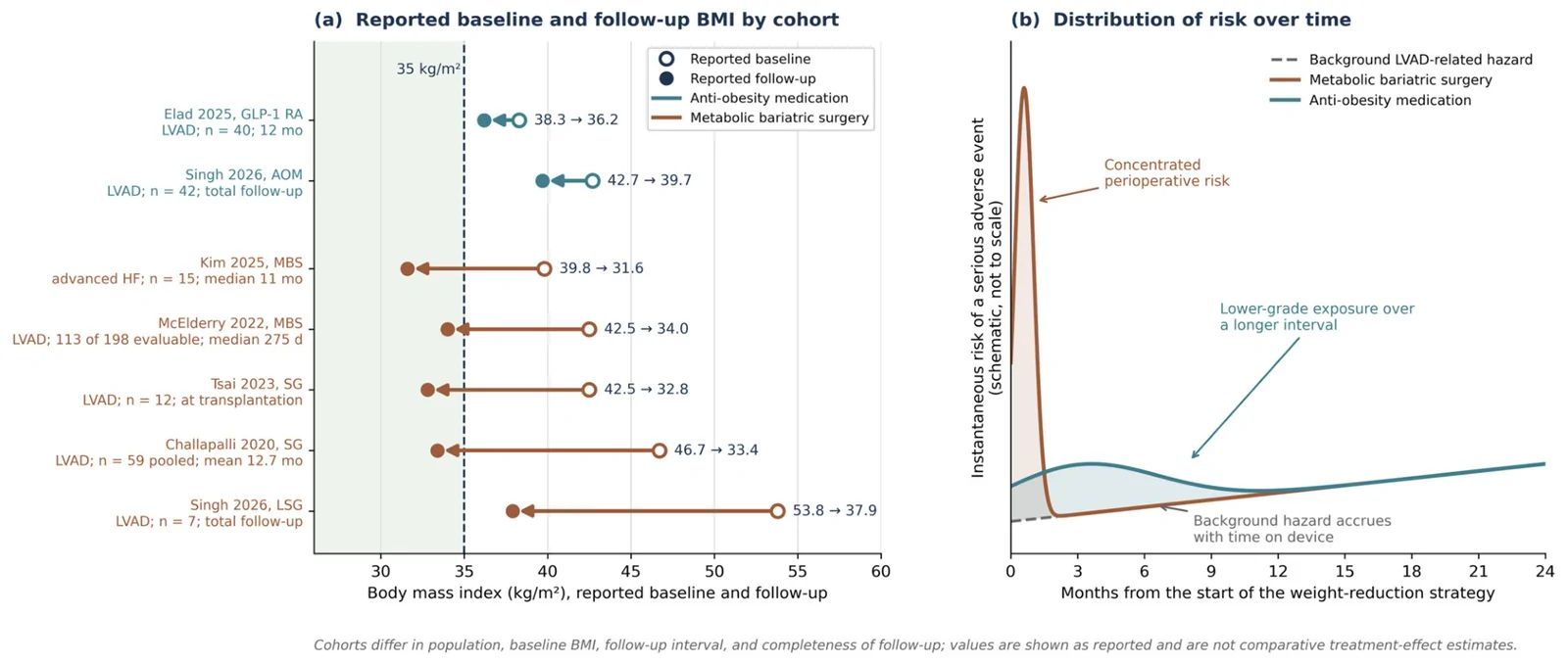
BMI trajectories reported by individual cohorts and the distribution of risk over time. (**a**) Reported baseline (open circles) and follow-up (filled circles) BMI in published cohorts [13,22,42,45,46,48] of LVAD-supported and advanced heart failure patients, with the dashed line marking 35 kg/m^2^. Values are shown as reported (medians or means) at differing follow-up intervals; populations, baseline BMI, and completeness of follow-up differ, and the McElderry values are based on the 113 of 198 patients with follow-up BMI. The panel is descriptive and does not provide comparative treatment-effect estimates. (**b**) Schematic representation of how risk is distributed over time. Metabolic bariatric surgery concentrates risk in the perioperative window and then reverts toward the background device-related hazard, whereas anti-obesity medications add a lower-grade exposure spread across a longer interval during which that background hazard continues to accrue. Panel (**b**) is conceptual and not drawn to scale. AOM, anti-obesity medication; BMI, body mass index; GLP-1 RA, glucagon-like peptide-1 receptor agonist; HF, heart failure; LSG, laparoscopic sleeve gastrectomy; LVAD, left ventricular assist device; MBS, metabolic bariatric surgery; SG, sleeve gastrectomy. Created in BioRender. Sicouri, S. (2026) https://BioRender.com/vvfnt9z.

**Table 1:** Published outcomes of anti-obesity medications and metabolic bariatric surgery in LVAD-supported and advanced heart failure populations. Values are shown as reported; cohorts differ in population, baseline BMI, follow-up interval, and completeness of follow-up, and they are not directly comparable.

Study (year)	Design and Population	Intervention	BMI or Weight Outcome	Transplant Outcome	Safety Outcome
**Anti-obesity medications**					
Elad et al., 2025 [22]	Single-center retrospective; 44 of 333 LVAD recipients (13.2%) started a GLP-1 RA; 40 (12.0%) analyzed, 2018–2023	GLP-1 RA (semaglutide 90%), 1 year	Weight 116 (98–134) → 110 (91–129) kg, *p* < 0.001; BMI 38.3 (33.3–45.4) → 36.2 (31.4–41.2) kg/m^2^, *p* < 0.001; 26% of the 27 patients with baseline BMI > 35 kg/m^2^ reached <35 kg/m^2^ (numerator not reported)	Not reported	HF hospitalization 6 (15.0%); driveline infection 5 (12.5%); HRAE 3 (7.5%); GI bleeding 2 (5.0%); 1 death (2.5%, device malfunction); no cannula angle change with weight loss; 3 (7.5%) stopped for cost or coverage, none for adverse events
Edwards et al., 2024 [23]	Single-center series; 21 LVAD recipients with WHO class I–III obesity	GLP-1 agonist, median 12 months (6–22)	Median weight −12.4 kg (−20.0 to −2.9); −7.4% of body weight; 17/21 lost weight	Not reported	9/21 required a pump speed reduction; no major adverse event attributed to therapy
Ibrahim et al., 2025 [24]	Propensity-matched federated network; 1036 LVAD recipients (518 matched pairs)	GLP-1 RA use vs. non-use	Not reported	Transplantation 18.9% vs. 8.5%; aOR 2.51 (95% CI, 1.72–3.67); observational association	Acute HF events 55.6% vs. 68.9% (aOR 0.57); all-cause hospitalization 62.5% vs. 75.3% (aOR 0.55); no difference in mortality, stroke, or cardiac arrest
Divya et al., 2026 [25]	Propensity-matched federated network; 527 obese LVAD recipients, 141 matched pairs, 2016–2024	Documented pre-implant GLP-1 RA exposure	Not reported	Higher rates of transplant listing and transplantation at 3 years; observational association	Lower hazard of driveline infection at 1 year; lower all-cause mortality at 3 years in exposed patients
**Metabolic bariatric surgery**					
Zenilman et al., 2019 [44]	Single-center case series; 6 LVAD recipients, 2014–2018	LSG with heparin bridging	Mean BMI 41.4 kg/m^2^ at surgery (range 37.1–58)	4 (67%) listed, 3 within 1 year; 3 (50%) transplanted	Median LOS 8.5 d; 2 (33%) readmitted without significant adverse outcome
McElderry et al., 2022 [42]	Medicare 2012–2019; 198 of 2798 LVAD recipients with ≥class II obesity	SG 93.9%; RYGB 6.1%	Median BMI 42.5 → 34 kg/m^2^, median loss 9.5 (IQR 5–14); 69/113 evaluable (61%) reached ≤35 (study definition) and 35/113 ≤ 30 kg/m^2^; follow-up BMI missing in 85/198 (43%)	37 (18.7%) transplanted at median 371 d (IQR 246–575); sHR 2.95 (95% CI, 2.09–4.17); 13 (6.6%) explanted for recovery	30-d MACE 6.1%; 1-y mortality 12.6%; HF readmission IRR 0.20 (95% CI, 0.11–0.38); no mortality association (HR 0.88; 95% CI, 0.67–1.14)
Tsai et al., 2023 [45]	Single-center; 12 LVAD recipients, 2018–2022	SG (9 robotic, 3 laparoscopic)	Mean BMI 42.5 before SG → 32.8 at transplantation; 66.7% reached <35 kg/m^2^	4 (33.3%) transplanted; mean 17.9 mo from SG to transplantation	1 reoperation for bleeding on POD 1; 1 readmission for hematochezia on POD 7
Kim et al., 2025 [46]	Single-center; 15 advanced HF with BMI > 35 kg/m^2^ vs. 62 without surgery	SG 12; RYGB 3	BMI 39.8 (39.0–42.2) → 31.6 (27.7–35.3) kg/m^2^ at median 11 mo, *p* < 0.001	Post-transplant survival comparable between groups	No surgery-related mortality
Goubar et al., 2025 [47]	Matched retrospective cohort; 20 HFrEF vs. 40 controls	SG	ΔBMI −9.9 (95% CI, −12.2 to −7.6) vs. −2.7 kg/m^2^, *p* < 0.05	Bridge to candidacy or recovery	Survival HR 0.20 (95% CI, 0.07–0.62); no deaths vs. 35% in controls
Challapalli et al., 2020 [48]	Meta-analysis of 8 studies; 59 LVAD recipients (22 concurrent, 37 staged)	SG	Mean BMI 46.7 (95% CI, 42.9–50.6) → 33.4 (95% CI, 30.2–36.6) kg/m^2^, *p* < 0.01	66% listed; 33% transplanted	No significant difference in complications or survival between concurrent and staged approaches; wide confidence intervals
Barajas-Gamboa et al., 2026 [33]	MBSAQIP 2023; 133 LVAD among 180,544 primary cases	SG 73.7%; remainder RYGB	Not reported	Not reported	Serious complications 11.3% vs. 2.5%; bleeding 5.3% vs. 0.8%; readmission 13.5% vs. 2.9%; LOS 4.9 vs. 1.2 d; adjusted OR 2.83 (95% CI, 1.62–4.97)
**Direct comparisons**					
Sher et al., 2023 [49]	Mixed retrospective and prospective; 18 patients with HF and BMI > 35 kg/m^2^, LVAD and non-LVAD	7 surgical vs. 11 non-surgical	6 mo: ΔBMI −6.4 vs. −0.7 kg/m^2^; weight −18.6 vs. −1.9 kg	Not reported	LVEF +5.9% vs. −5.9%, not significant
Singh et al., 2026 [13]	Single-center; 49 LVAD recipients	42 AOM (semaglutide, tirzepatide, dulaglutide) vs. 7 LSG	Baseline BMI 42.7 vs. 53.8 kg/m^2^; 3-mo ΔBMI −0.9 vs. −8.6; total −3.0 vs. −15.9 kg/m^2^	Listed 5/42 vs. 1/7; transplanted 3/42 vs. 1/7	3 deaths from HF progression; 1 postoperative death within 6 mo

AOM, anti-obesity medication; BMI, body mass index; CI, confidence interval; GLP-1 RA, glucagon-like peptide-1 receptor agonist; HbA1c, glycated hemoglobin; HF, heart failure; HFrEF, heart failure with reduced ejection fraction; HR, hazard ratio; HRAE, hemocompatibility-related adverse event; aOR, adjusted odds ratio; IQR, interquartile range; IRR, incidence rate ratio; LOS, length of stay; LSG, laparoscopic sleeve gastrectomy; LVAD, left ventricular assist device; LVEF, left ventricular ejection fraction; MACE, major adverse cardiovascular events; MBSAQIP, Metabolic and Bariatric Surgery Accreditation and Quality Improvement Program; OR, odds ratio; POD, postoperative day; RYGB, Roux-en-Y gastric bypass; SG, sleeve gastrectomy; sHR, subdistribution hazard ratio.

**Table 2:** Domain-by-domain comparison of anti-obesity medications and metabolic bariatric surgery in LVAD-supported patients, with the source of evidence for each domain.

Domain	Anti-Obesity Medications	Metabolic Bariatric Surgery	Evidence Base
Principal mechanism	Central and peripheral suppression of caloric intake; delayed gastric emptying	Reduced gastric reservoir plus enteroendocrine shift; ghrelin suppression after sleeve gastrectomy	General obesity; mechanistic studies
Reported BMI reduction in LVAD or advanced HF cohorts	Approximately 2 kg/m^2^ over 12 months; 26% of the 27 patients who began above 35 kg/m^2^ reached <35 kg/m^2^ at 1 year (numerator not reported)	Approximately 6–10 kg/m^2^ over 6–12 months; 69 of 113 evaluable Medicare patients (61%) reached ≤35 kg/m^2^; follow-up BMI missing in 85 of 198 (43%), so at least 35% of the full cohort	LVAD-specific, observational [22,42]
Time to reach a BMI < 35 kg/m^2^	Feasible only for patients close to the threshold; may exceed the interval of support that is well tolerated otherwise	Typically achieved within 6–12 months across published series	LVAD-specific, observational; expert extrapolation
Acute procedural risk	Not applicable; no procedural intervention. Treatment-related risks (gastrointestinal intolerance, volume depletion) are not absent	Serious 30-day complications in 11.3% of LVAD recipients; bleeding in 5.3%	LVAD-specific, national registry [33]
Distribution of risk	Low-grade exposure spread across a prolonged interval, added to accruing device hazard	Concentrated in the perioperative window, then reverting toward background device hazard	Expert extrapolation
Dominant LVAD-specific hazard	Volume depletion and preload reduction with a theoretical risk of suction events; non-significant, numerically unfavorable signal with liraglutide in non-LVAD advanced HFrEF; cannula position did not shift with weight loss in the one study that measured it	Bleeding under therapeutic anticoagulation; marginal ulceration after gastric bypass; risk highest when performed during the implantation admission	LVAD-specific [22,33,42]; advanced HF RCT [19]; general bariatric [35,36,37,38]
Heart failure events and hemodynamics	Fewer acute HF events (aOR 0.57) in a matched network cohort; invasive hemodynamics unchanged in 40 patients; pump speed reduced in 9 of 21	HF readmissions 3.01 → 0.62 per 100 person-days; 6.6% explanted for recovery; no hemodynamic data	LVAD-specific, observational [22,23,24,42]
Arrhythmic outcomes	No LVAD data on ventricular arrhythmia or atrial fibrillation; cardiac arrest 3.5% vs. 3.3% (not significant); outside LVAD support, atrial fibrillation incidence neutral or lower	No LVAD data; outside LVAD support, atrial fibrillation not significantly reduced (HR 0.82; 95% CI, 0.64–1.06)	LVAD-specific for cardiac arrest only [24]; extrapolated [53,54,55]
Right ventricular function	Not reported	Not reported	No data; evidence gap
Cardiovascular events outside LVAD support	MACE 6.5% vs. 8.0% with semaglutide (HR 0.80; 95% CI, 0.72–0.90)	Lower all-cause and cardiovascular mortality and incident HF in observational cohorts	Extrapolated: RCT [52]; meta-analysis of mainly observational data [55]
Anticoagulation implications	Altered oral absorption from delayed gastric emptying; closer INR surveillance during titration	Reduced time in therapeutic range for up to 6 months and altered warfarin dose requirements after sleeve gastrectomy	LVAD-specific for surgery [41]; mechanistic for medications [21]
Durability	Dependent on continued adherence and access; regain expected after discontinuation	More durable than pharmacologic weight loss after discontinuation; maintenance still depends on nutritional, behavioral, and clinical follow-up, and weight regain can occur	General obesity and bariatric [50]; expert extrapolation
Leading cause of treatment failure	Cost and insurance non-coverage; 3 of 40 (7.5%) discontinued for this reason in the largest LVAD series	Insufficient weight loss or a plateau above the threshold	LVAD-specific [22]; expert extrapolation
Reversibility	Fully reversible on discontinuation	Anatomically irreversible	Intrinsic to the intervention
Association with transplant access	Transplantation 18.9% vs. 8.5% in a propensity-matched network cohort (aOR 2.51; 95% CI, 1.72–3.67); observational, confounding likely	Threefold higher probability of transplantation (sHR 2.95; 95% CI, 2.09–4.17); no associated change in mortality; observational, confounding likely	LVAD-specific, observational [24,42]
Strength of LVAD-specific evidence	Four observational cohorts: two single-center series and two propensity-matched analyses of the same federated network	One national database analysis, one Medicare cohort, a meta-analysis of 59 patients, and several single-center series	Observational only; no randomized data

**Table 3:** Hypothesis-generating framework, based on expert opinion, for considering the initial strategy.

Consideration	May Favor Consideration of a Pharmacologic Trial First	May Favor Consideration of Early Surgical Referral	Evidence Source
Excess BMI above the 35 kg/m^2^ threshold	Within approximately 3–4 kg/m^2^ (illustrative; extrapolated from an approximately 2 kg/m^2^ annual reduction)	More than approximately 5 kg/m^2^ above the threshold (illustrative)	LVAD-specific, observational [22,42]; expert extrapolation
Anticipated risk of device-related complications	Recent implantation, no hemocompatibility events, stable driveline	Prior bleeding, infection, or pump thrombosis; long-standing support	LVAD registry data [7,8]; expert extrapolation
Interval since LVAD implantation	Still within the index admission or the early recovery period	Recovered from implantation and stable; staged surgery in the Medicare cohort was performed at a median of 702 days after implantation	LVAD-specific, observational [42,43]
Bleeding and anticoagulation profile	Prior gastrointestinal bleeding, recurrent hemocompatibility-related adverse events, difficult INR control	Stable anticoagulation, no bleeding history, no prior abdominal surgery	LVAD-specific [33,41]; general bariatric [32,34]
Gastrointestinal tolerance and nutritional status	Preserved appetite and nutritional reserve	Established gastrointestinal intolerance of incretin therapy or failed pharmacologic trial	Expert extrapolation
Right ventricular and hemodynamic reserve	Marginal right ventricular function, marked preload dependence, prior suction events	Preserved right ventricular function and stable hemodynamics	Mechanistic [20]; no LVAD outcome data
Recent ventricular arrhythmia or electrical storm	Stabilize rhythm before starting; volume depletion during titration may provoke suction-related arrhythmia	Stabilize rhythm before referral; perioperative arrhythmic risk not quantified in LVAD recipients	Mechanistic and advanced HF data [20,51]; expert extrapolation
Adherence, access, and coverage	Reliable adherence and drug coverage confirmed in advance	Uncertain coverage, poor adherence, or anticipated interruption of therapy	LVAD-specific [22]
Institutional experience	Limited local bariatric experience with mechanical circulatory support	Established multidisciplinary bariatric and mechanical circulatory support program	2024 ISHLT guideline [4]; expert opinion

## Data Availability

No new data were created or analyzed in this study. All data discussed are available in the cited publications.

## Abbreviations

The following abbreviations are used in this manuscript:

AOM	Anti-obesity medication
BMI	Body mass index
GLP-1 RA	Glucagon-like peptide-1 receptor agonist
HF	Heart failure
HFrEF	Heart failure with reduced ejection fraction
HT	Heart transplantation
ISHLT	International Society for Heart and Lung Transplantation
LSG	Laparoscopic sleeve gastrectomy
LVAD	Left ventricular assist device
MBS	Metabolic bariatric surgery
MBSAQIP	Metabolic and Bariatric Surgery Accreditation and Quality Improvement Program
RYGB	Roux-en-Y gastric bypass
SG	Sleeve gastrectomy
